# Supplementary material for: Synergistic Antioxidant Effects of C3G-Enriched Oryza sativa L. cv. RD83 Extract and α-Tocopherol Against H2O2-Induced Oxidative Stress in SH-SY5Y Cells
Source: Int J Mol Sci. 2025 Jul 5;26(13):6490. doi: 10.3390/ijms26136490 (PMC12250117; doi:10.3390/ijms26136490)
Supplement: Supplementary file 1 [file ijms-26-06490-s001.zip › ijms-3701934-supplementary.pdf]

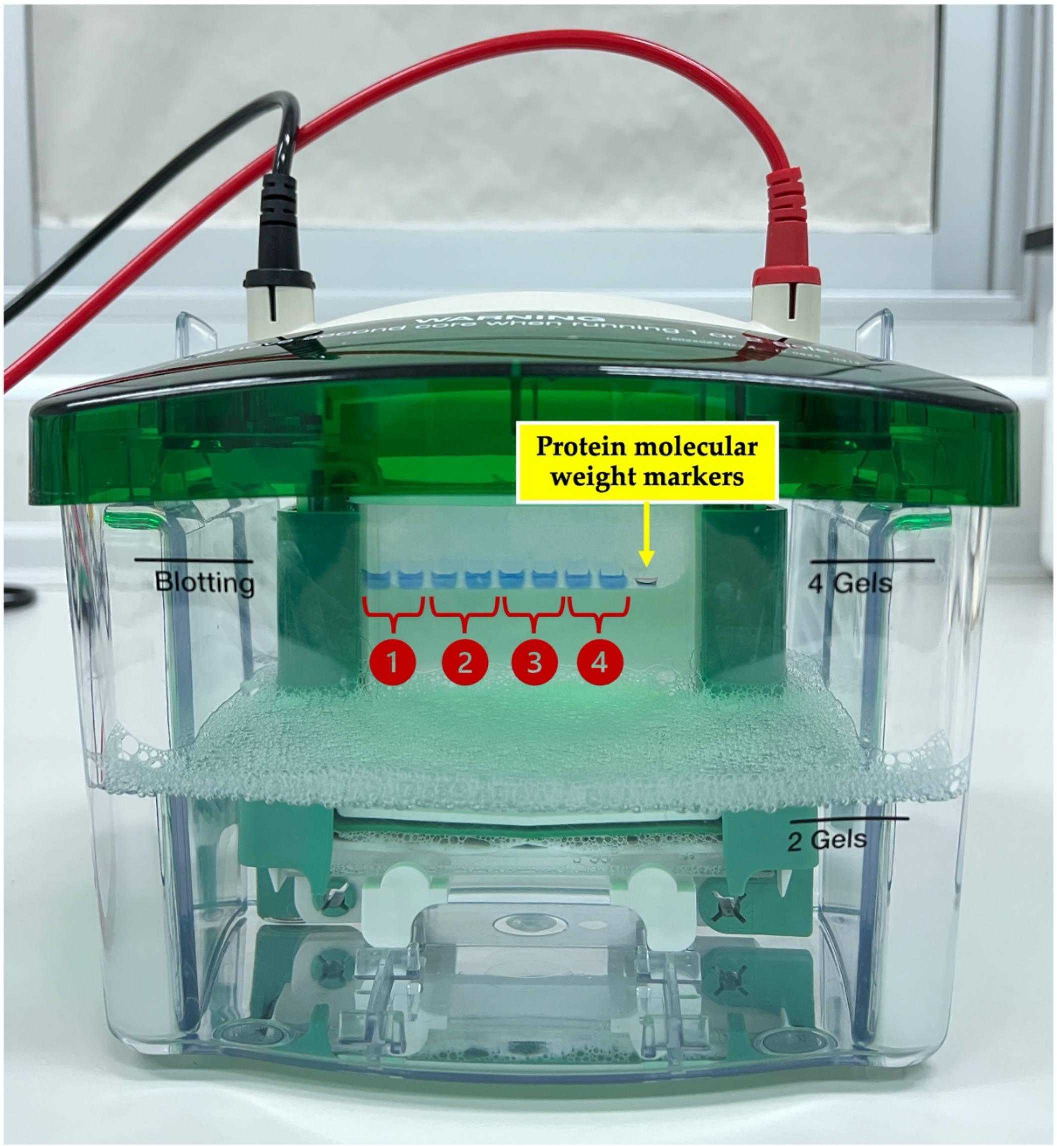

Figure S1: Representative Western blot analysis showing protein loading through the stacking gel and separation in the resolving gel. Each experiment was performed in duplicate. Experimental groups included: (1) naïve control, (2) H_2O_2 + vehicle, (3) H_2O_2 + C3GE (20 µg/mL), and (4) H_2O_2 + C3GE (40 µg/mL).

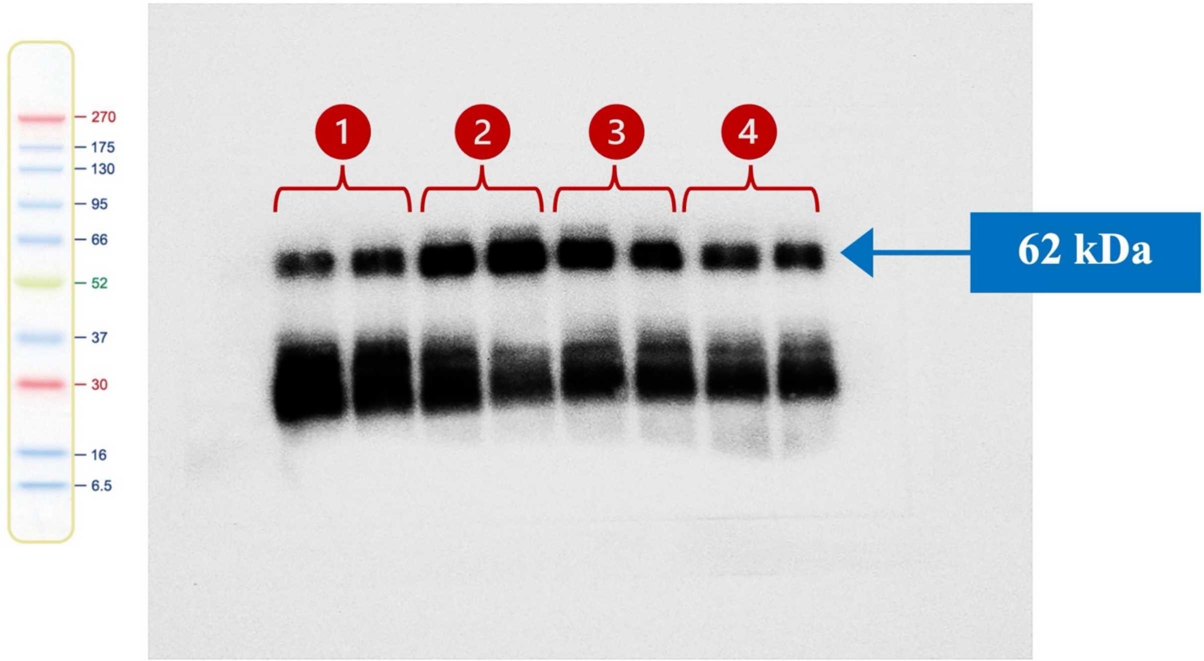

Figure S2: Full-length, uncropped Western blot membranes showing HDAC1 expression in SH-SY5Y cells subjected to hydrogen peroxide-induced oxidative stress and treated with C3GE. The experimental conditions were as follows: (1) untreated control, (2) H_2O_2 + vehicle, (3) H_2O_2 + C3GE (20 μg/mL), and (4) H_2O_2 + C3GE (40 μg/mL). The blots represent original, unprocessed data obtained from the experiment.

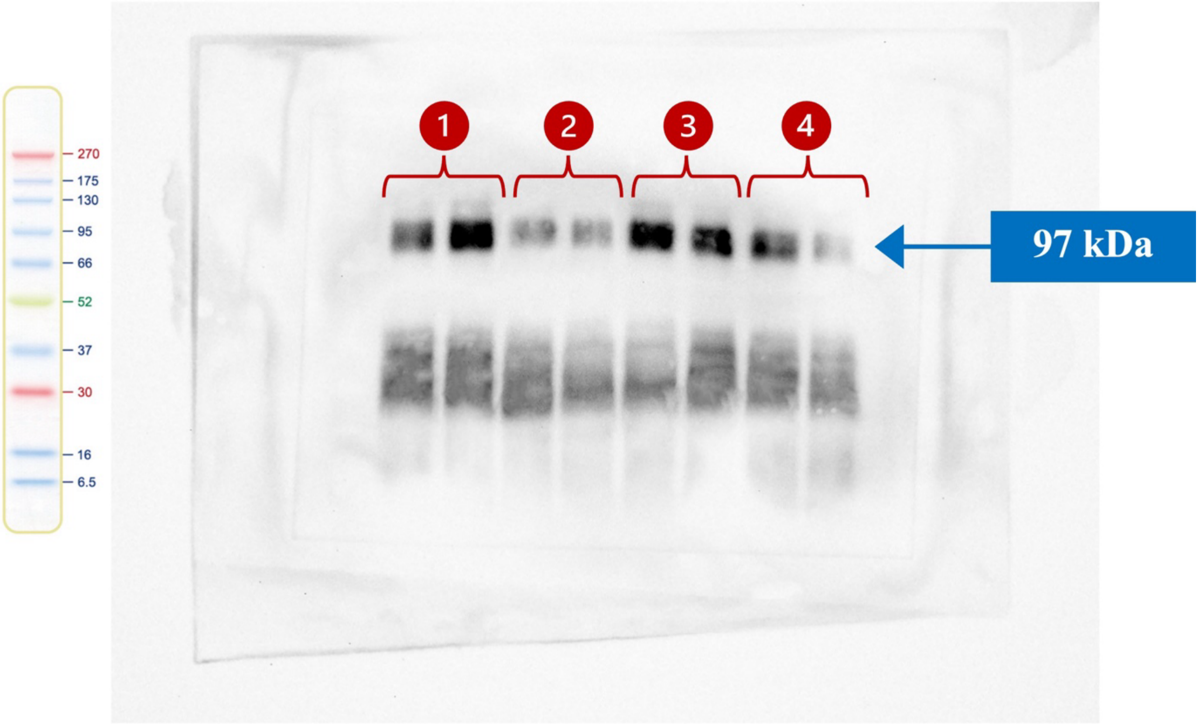

Figure S3: Full-length, uncropped Western blot membranes showing Nrf2 expression in SH-SY5Y cells subjected to hydrogen peroxide-induced oxidative stress and treated with C3GE. The experimental conditions were as follows: (1) untreated control, (2) H_2O_2 + vehicle, (3) H_2O_2 + C3GE (20 µg/mL), and (4) H_2O_2 + C3GE (40 µg/mL). The blots represent original, unprocessed data obtained from the experiment.

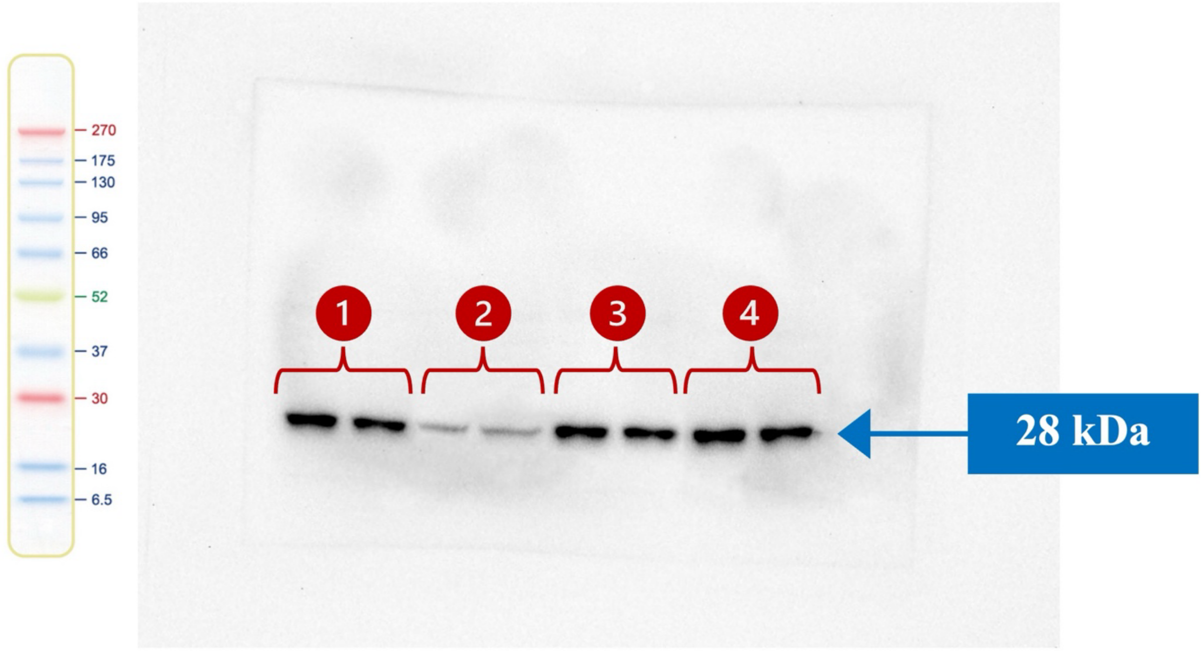

Figure S4: Full-length, uncropped Western blot membranes showing HO-1 expression in SH-SY5Y cells subjected to hydrogen peroxide-induced oxidative stress and treated with C3GE. The experimental conditions were as follows: (1) untreated control, (2) H_2O_2 + vehicle, (3) H_2O_2 + C3GE (20 μg/mL), and (4) H_2O_2 + C3GE (40 μg/mL). The blots represent original, unprocessed data obtained from the experiment.

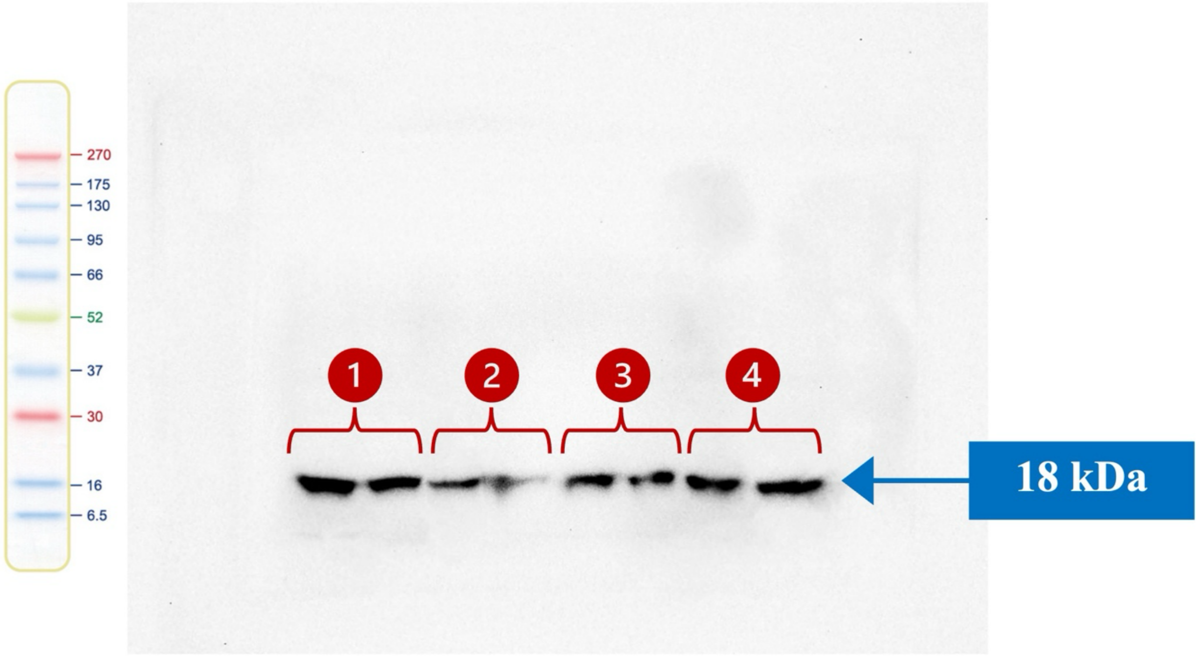

Figure S5: Full-length, uncropped Western blot membranes showing SOD1 expression in SH-SY5Y cells subjected to hydrogen peroxide-induced oxidative stress and treated with C3GE. The experimental conditions were as follows: (1) untreated control, (2) H_2O_2 + vehicle, (3) H_2O_2 + C3GE (20 μg/mL), and (4) H_2O_2 + C3GE (40 μg/mL). The blots represent original, unprocessed data obtained from the experiment.

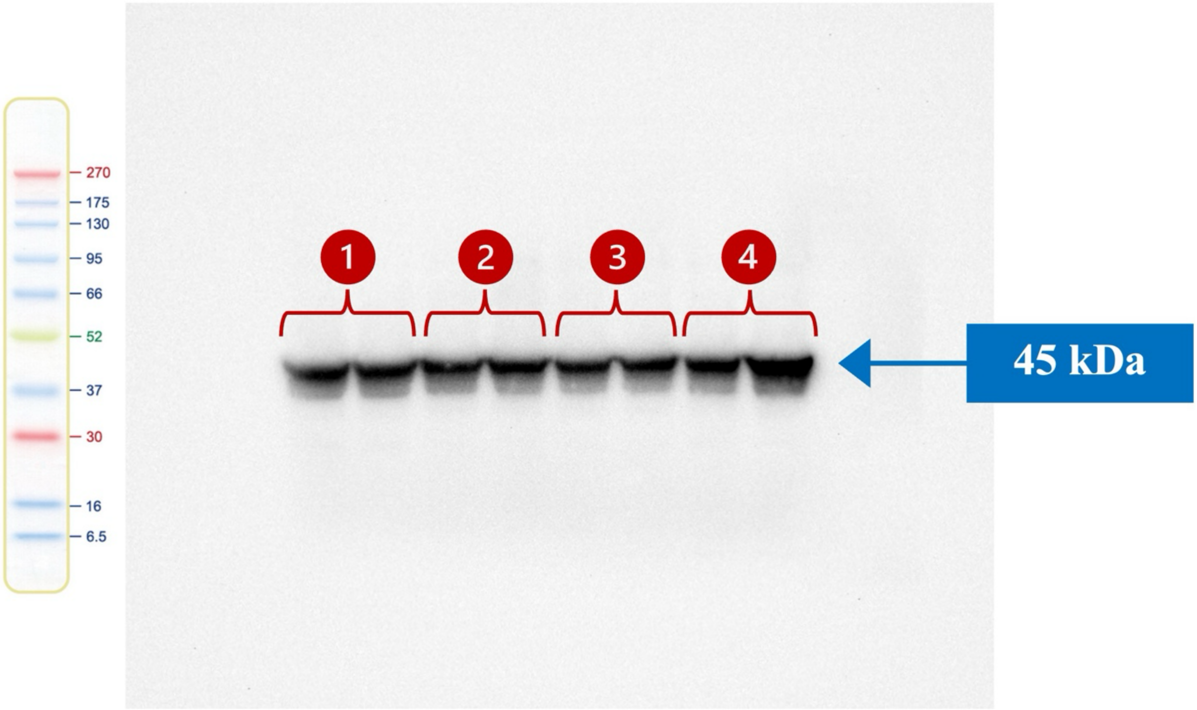

Figure S6: Uncropped Western blot image of β-actin expression in SH-SY5Y cells used as a loading control for normalization. The same experimental groups were applied: (1) untreated control, (2) H_2O_2 + vehicle, (3) H_2O_2 + C3GE (20 µg/mL), and (4) H_2O_2 + C3GE (40 µg/mL). The blots represent original, unprocessed data obtained from the experiment.
